# Eigenvector Centrality Dynamics From Resting-State fMRI: Gender and Age Differences in Healthy Subjects

**DOI:** 10.3389/fnins.2019.00648

**Published:** 2019-06-27

**Authors:** Alle Meije Wink

**Affiliations:** Radiology and Nuclear Medicine, Amsterdam University Medical Center, Amsterdam, Netherlands

**Keywords:** graph theory – graph algorithms, trees, functional MRI (fMRI) methods, imaging studies, age-related, gender-related

## Abstract

With the increasing use of functional brain network properties as markers of brain disorders, efficient visualization and evaluation methods have become essential. Eigenvector centrality mapping (ECM) of functional MRI (fMRI) data enables the representation of per-node graph theoretical measures as brain maps. This paper studies the use of centrality dynamics for measuring group differences in imaging studies. Imaging data were used from a publicly available imaging study, which included resting fMRI data. After warping the images to a standard space and masking cortical regions, ECM were computed in a sliding window. The dual regression method was used to identify dynamic centrality differences inside well-known resting-state networks between gender and age groups. Gender-related differences were found in the medial and lateral visual, motor, default mode, and executive control RSN, where male subjects had more consistent centrality variations within the network. Age-related differences between the youngest and oldest subjects, based on a median split, were found in the medial visual, executive control and left frontoparietal networks, where younger subjects had more consistent centrality variations within the network. Our findings show that centrality dynamics can be used to identify between-group functional brain network centrality differences, and that age and gender distributions studies need to be taken into account in functional imaging studies.

## Introduction

Functional brain network properties are increasingly studied as markers of neurological and psychiatric disorders. With the availability of fast computer hardware and increased memory, analyses have been focusing on the dynamics of networks. We describe an efficient implementation and application of dynamic centrality and its differences between males and females.

Resting functional magnetic resonance imaging (RfMRI) acquires MR images of the brain that are sensitive to blood oxygenation, which serves as a proxy for local brain activity. In the absence of a task, a number of recurring patterns in RfMRI data (Smith et al., 2009) are referred to as resting-state networks (RSNs) computed from pairwise similarity matrices between voxel time series (Damoiseaux et al., 2006). In the case of these non-evoked, non-causal, undirected similarities, the term ‘functional connectivity’ is used, and (Pearson) correlation is a common connectivity measure.

Visualization and interpretation of complete network connections matrices is increasingly problematic with increasing network size. Voxelwise whole-brain network analyses therefore focus on properties of network nodes. The concept of centrality of a node in a network represents the proportion of the network traffic involving that node. It can be expressed in a number of ways, for example:

– the number of connections of a node (degree centrality)
– the number of times a node appears in the shortest path between two other nodes (betweenness centrality).

For eigenvector centrality EC, this measure is the coefficient in the dominant eigenvector (the one with the largest eigenvalue) of the connections matrix. It can be computed iteratively as the weighted sum of centralities of a node’s neighbors. Voxelwise EC computation, or eigenvector centrality mapping (ECM) (Lohmann et al., 2010), has shown to provide a measure for functional brain network analysis that is robust with respect to physiological and technical confounding factors and sensitive to changes correlated with pathology.

Eigenvector centrality mapping has been used to identify functional brain network changes in patients, e.g., with Alzheimer’s disease (AD) (Binnewijzend et al., 2014), multiple sclerosis (Eijlers et al., 2017), and healthy subjects at risk for AD (Wink et al., 2018).

The observation that functional brain properties vary over time led to the emergence of dynamic functional connectivity (Tagliazucchi et al., 2016; Preti et al., 2017). Unlike dynamic causal modeling experiments, where a change in cognitive tasks leads to a different connection pattern, dynamic functional connectivity itself is also non-evoked and non-causal. Functional similarities such as the correlation between time series, are measured inside a sampling interval, and network dynamics are often measured by shifting a fixed-size time window across the scan duration. The result is a time series of network measures which can be further analyzed.

This makes dynamic functional connectivity as a method bit of a double-edged sword: properties of temporal connectivity can be studied in greater detail, but the method produces an enormous amount of intermediate results, requiring an extra step in analysis, visualization, and interpretation.

We propose dynamic eigenvector centrality mapping (dECM), which produces a time series of centrality maps, each of which is an image of the same size as the original volumes. This allows easy and intuitive visualization of each window position for which centrality is computed. This technique has been used before as an intermediate result (Preti and Ville, 2017), which showed that dominant EC patterns over time are similar to RSN. This paper uses those previous findings to detect group-based differences in EC variability.

We use dual regression, an analysis used for functional connectivity studies (Nickerson et al., 2017), to demonstrate how differences between males and females and age groups can be found in a public example data set.

## Materials and Methods

### Data From OpenNeuro

Imaging data was downloaded from the OpenNeuro project^1^. The “Washington 120” data set includes structural T1-weighted MR images and resting fMRI time series of 120 healthy subjects (59 male), recruited from Washington University. Subjects were right-handed native English speakers, reported no neurological or psychiatric disease, were not on medication and had previously given written informed consent (Power et al., 2013). Mean age was 24.7 years at scanning (median: 24.5) and standard deviation 2.4 years (interquartile range: 3.1). Ages were not significantly different between males and females (Kruskal–Wallis *p* = 0.5).

Imaging was done on a Siemens MAGNETOM Trio 3T scanner (Erlangen, Germany) with a Siemens 12 channel Head Matrix Coil. The T1-weighted scans used the MP-RAGE sequence with TE = 3.06 ms, TR-partition = 2.4 s, TI = 1000 ms, flip angle = 8° and 127 slices with 1 mm × 1 mm × 1 mm voxels. Resting fMRI (RfMRI) was acquired using an EPI sequence with TE = 27 ms, TR = 2.5 s, flip angle = 90° and 32 contiguous interleaved 4 mm axial slices with in-plane resolution = 4 mm × 4 mm. Subjects kept their eyes open during scanning, fixating on a cross displayed on the screen. The duration of scan varied from 130 volumes ∼ 5 min, to 720 volumes ∼ 30 min.

The OpenNeuro website provides the preprocessed data, using SPM^2^ version 8 (Ashburner and Friston, 2005), in standard space. The original scans had been corrected for slice timing to align the start times of all slices per volume, spatially realigned to correct head motion, and their intensities normalized to a mean of 1000. Images were then resampled to MNI-space on an isotropic 3 mm grid (Power et al., 2014). The preprocessing using mriqc (Esteban et al., 2017) reported five subjects with a mean frame displacement FD (Power et al., 2012) of 0.2 mm. We excluded these five subjects from our analysis. Remaining motion-related effects were removed from the standard-space time series by computing the effects of motion using single-subject GLMs with the motion parameters as covariates, and subtracting these from the data (Soares et al., 2016). The resulting data set were 115 subjects (57 male) with median age 24.5 years (57 below) whose time series had the linear effects of rigid-body motion parameters for removed for each volume.

The T1-weigted images available from the OpenNeuro website were accompanied by segmentations into tissue types and parameters for mapping those to the MNI space in SPM. We applied these parameters to the anatomical images to bring them in the same space as the RfMRI data.

The gray matter masks were thresholded at 20% gray matter density and binarized before being mapped into MNI space. The MNI-space masks were then thresholded again at 0.2 and binarized, to create one gray matter mask for the group. A mask of each of the functional images was created by taking the time series’ minimum at each voxel, and thresholding the map of temporal minima at the 20^th^ percentile, yielding a binary mask. The group mean over all subjects’ binary masks was thresholded at 75% yielding one binary mask for the group. Every RfMRI data set was masked by the intersection of the group gray matter and functional masks and an additional mask to exclude the cerebellum. Using the same mask for every subject ensured that sizes between the RfMRI networks did not vary, which would have been a source of variance (Van Wijk et al., 2010).

### Eigenvector Centrality Mapping

Most connectivity analyses for RfMRI are based on the matrix ***R*** of pairwise correlations between voxels or brain regions. In the voxelwise case, the size of this matrix poses two problems: (i) the matrix is too big for the working memory of standard computers and (ii) the total connectome, i.e., the set of connections describing a condition or an experiment, is to big to visualize or interpret. A common solution is to spatially downsample the data or to group the voxels into sub-networks or parcellations. Another approach is to look at node properties instead of connections, with the benefit of keeping the full imaging resolution. The centrality property signifies the prominence of a node in terms of the participation in the connections of the network.

Centrality can be expressed using a growing number of measures. The simplest is degree: the number of connections of a node to others. One of the more intuitive measures of a node’s centrality is betweenness: the proportion of all the shortest paths between pairs of nodes in the network on which it lies (Fletcher and Wennekers, 2016).

A node’s eigenvector centrality (EC) is defined as the sum of its neighbors’ centralities (Lohmann et al., 2010) and can be efficiently computed from the connections matrix ***R*** using power iteration if this matrix is semi-definite and positive (or irreducible and non-negative). Power iteration starts with an estimate vector *v*_0_ and computes

(1) $$v_{i+1}\;=\;R^{*}v_{i}$$

followed by *L*_2_ normalization, for subsequent steps, until a convergence criterion is met. A computationally efficient definition of voxelwise ECM uses ***R***+1 as the connections matrix to ensure positivity and the fact that for intensity-normalized scaled fMRI data ***Y**_[N × T]_* of *N* voxels and *T* time points (Equation 1) can be re-written as

(2) $$v_{i+1}\;=\;Y_{\left[N\times T\right]}\;*\;\left(Y^{T}{}_{\left[T\times N\right]}\;*\;v_{i\left[N\times 1\right]}\right)$$

so that it needs to store at most *NT* values during computation instead of *N*^2^ (Wink et al., 2012). The ECM technique has since been used in a number of settings to differentiate between experimental conditions and patient groups (Lohmann et al., 2010; Wink et al., 2012, 2018; Binnewijzend et al., 2014; Schoonheim et al., 2014; Duinkerken et al., 2017; Eijlers et al., 2017).

Correlations of signals measured during a time interval are static measures, yet we know that functional connections in the brain are dynamic in that their strength varies in time (Tagliazucchi et al., 2012; Zalesky and Breakspear, 2015; Breakspear, 2017). To capture the temporal evolution of centrality, we computed voxelwise EC in a sliding window, resulting in a time series of 3-dimensional EC maps. We used the fastECM matlab toolbox^3^ for ECM computations, which enables the computation of centrality time series as follows: given the number *T* of volumes in the input time series and a requested number *M* of centrality maps, it moves a window of *T-M+1* volumes over all *M* different positions and returns the time series of centrality maps. For the data used in this paper, with input time series of different lengths, this meant that the size of the window for EC computation varied between subjects.

**Figure 1:**
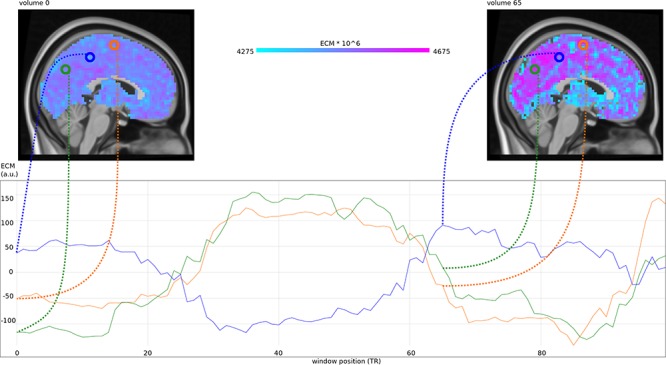
**(Top)** Eigenvector centrality maps (ECM) computed in a sliding window at 100 different positions, at window position 0^∗^TR (a) and 65^∗^TR (b). **(Bottom)** Mean-centered centrality timecourses for voxels at position (3, –67, 35) in MNI space (green), position (3, –37, 50) in MNI space (blue) and position (3, –7, 65) in MNI space (orange).

We used *M* = 100, resulting in a time series of 100 ECMs per subject (see Figure 1), containing each voxel’s centrality given the starting time of the interval in which it was computed. Maps were multiplied to have intensities around 4500.

### Statistics of EC Variability Over Time

Each map of the EC time series shows the spatial pattern of the voxelwise temporal correlations inside its time window, and if well-defined RSNs such as the default mode network (DMN) and visual networks (Damoiseaux et al., 2006) vary over time, these variations should be visible in the time-varying EC maps. One way to determine this temporal variability is using dual regressions, DR (Nickerson et al., 2017). This method first regresses each fMRI data set onto a set of spatial patterns, e.g., ones that represent RSN, resulting in a representative time series per pattern, per subject. After that, the fMRI data are regressed onto these representative time series, yielding a voxelwise measure of temporal correspondence with each pattern, for each subject. Both steps are generalized linear models: for data ***Y**_[N × T]_* and a set ***C**_[N × M]_* of *M* spatial patterns, the first step solves

(3) $$Y_{\left[N\times T\right]}\;=\;C_{\left[N\times M\right]}\;*\;{\text{S}}_{\left[M\times T\right]}\;+\;{\text{ε}}_{\left[N\times T\right]}$$

for the pattern-specific time series ***S**[M × T]*, and the next step solves

(4) $$Y_{\left[N\times T\right]}\;=\;D_{\left[N\times M\right]}\;*\;S_{\left[M\times T\right]}\;+\;{\text{ε}}_{\left[N\times T\right]}$$

for the voxelwise correspondence ***D**_[N × M]_* to ***S**_[M × T]_*, and can be computed with a least squares method. The dual regression method, first introduced in 2009 (Beckmann, 2009) has been used to identify between-group RSNs differences (Smith et al., 2014; Nickerson et al., 2017). We computed DR using 10 spatial components linked to RSN by performing independent component analysis (ICA) on the results of 7,342 fMRI analyses (Smith et al., 2009), resulting in 10 regression maps per subject between-group differences in temporal EC variations between female and male participants, and young vs. old participants, respectively, were assessed for each RSN mask except the ‘cerebellar’ RSN with permutation statistics, using 10000 permutations and family-wise error (FWE) control with threshold-free cluster enhancement (TFCE) and a significance threshold of *p* < 0.05. FWE was done by re-computing the statistics (Equation) after group/age permutation, respectively and storing the maximum statistic found (Nichols and Holmes, 2002). Observed statistics were compared with the histogram of these maximum statistics, thus achieving location independence, and therefore, FWE control. TFCE (Smith and Nichols, 2009) increases the statistic values of each peak that is part of a spatial cluster by integrating the cluster’s spatial extent at different levels below the peak, providing a balance between correction for multiple voxelwise tests and relaxing the correction for voxels that are part of a cluster (and therefore not independent). Effects of gender and age were investigated in the between-group design using a contrast between male and female participants and younger and older participants, respectively, the latter being defined by a median split. A covariate for scan length (and thus, window length) was also included in the model. The dual regression script was slightly modified to only test for EC variations with each RSN pattern inside the mask (of Z-scores for that pattern above Section “Statistics of EC Variability Over Time”), as used previously (Binnewijzend et al., 2012). Analysis scripts for this paper are available at https://github.com/amwink/openNeuro.

## Results

### Participant Sub-groups

The median(mean) age of the female and male participants, respectively, were 24.4(24.5) and 24.9(24.5), and their standard deviations (interquartile ranges) were 1.9(3.1) and 2.6(3.2), respectively. The median split of the subjects into younger and older resulted in a ‘younger’ and ‘older’ group with mean (median) age of 22.8(23.2) and 26.5(26.3), respectively, and standard deviations (interquartile ranges) of 1.1(1.3) and 1.5(2.1), respectively. The ‘younger’ and ‘older’ group both consisted of 28 males and 29 females. The distributions of ages did not differ significantly for males and females in the young subjects (Kruskal–Wallis *p* = 0.39; it did so in the old subjects (Kruskal–Wallis *p* = 0.04) where the males were older than the females. Across the whole group there was no effect (Kruskal–Wallis *p* = 0.32).

### Eigenvector Centrality Mapping

**Figure 2:**
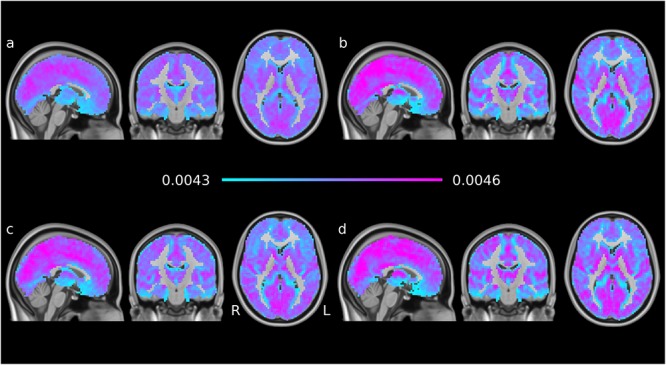
EC time series means, averaged per subgroup: **(a)** younger males, **(b)** younger females, **(c)** older males, **(d)** older females. The groups are generally similar; the female participants show more contrast than the males participants and the older groups show higher peaks than the younger groups of the same gender.

Time averages of each subject’s voxelwise EC time series were combined for four subgroups: young-old × male-female. The averages across these groups are shown in Figure 2 The overall features of the maps are very similar in the groups and to individual EC maps per volume (see Figure 1), indicating robust features and temporal stability.

### Statistics of EC Variability Over Time

**Figure 3:**
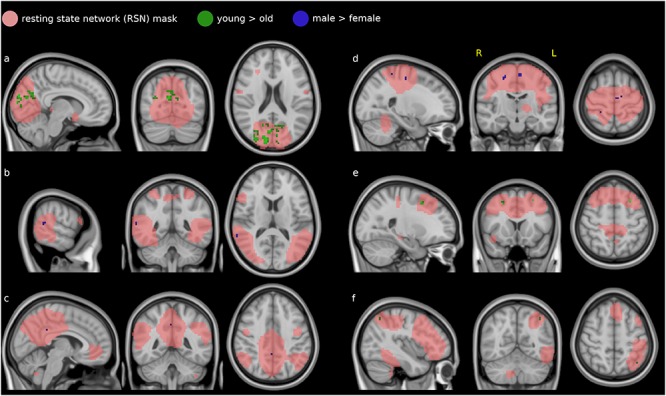
Difference in covariability of EC with established resting-state networks (RSN, salmon) between males and females (blue) and younger and older subjects (green): the medial visual network **(a)**, the lateral visual network **(b)** the default mode network **(c)**, the sensorimotor network **(d)**, the executive control network **(e)**, and the left fronto-parietal network **(f)**.

The dual regression analysis and non-parametric testing showed effects for a number of resting network patterns. Many of these were limited to single voxels; we report only the clusters of more than 10 voxels. These were found in the medial and lateral visual networks (see Figure 3a,b), the default mode network (Figure 3c), the sensorimotor network (Figure 3d), the auditory network (Figure 3e), and the left fronto-parietal network (Figure 3f). The EC variations in the other networks (occipital visual, lateral visual, cerebellum, executive control and right fronto-parietal) did not differ between male and female subjects or between the two age groups.

In the medial visual network (Figure 3a), EC covariations with the mean time course were lower in females than in males (blue voxels) in the precuneus. In a partly overlapping region they were also lower in the older group than the younger (green voxels). This region was concentrated in the cuneus and precuneus, touching visual cortices V1 (Brodmann area 18) and, to a lesser extent, V2 (Brodmann area 17).

Two small clusters in the lateral visual network (Figure 3b) showed lower correlations in the female group compared to male (blue voxels). These voxels were in the right medial temporal gyrus and bilateral inferior temporal gyrus.

In the default mode network, DMN (Figure 3c), two voxel locations showed lower covariatons in EC with its mean time course in the female group compared to the male (blue voxels). These voxels were contained in the parietal lobe: precuneus and posterior cingulate cortex.

EC covariations with the sensorimotor network (Figure 3d) were lower in females than in males (blue voxels) in a small region in the primary motor cortex (Brodmann area 4) and premotor cortex (Brodmann area 6) and primary somatosenory cortex (BA 2).

Smaller clusters were also found in the executive control network (Figure 3e), with lower covariations in females than males in the right medial frontal gyrus (blue voxels) and lower covariations in the older group than the younger in the left and right middle and superior frontal gyrus (green voxels).

In the left frontoparietal network (Figure 3f), EC covariations with the mean time course were lower in the older group than the younger in the left lateral occippital cortex (green voxels). No corresponding clusters were found in the right frontoparietal network.

**Figure 4:**
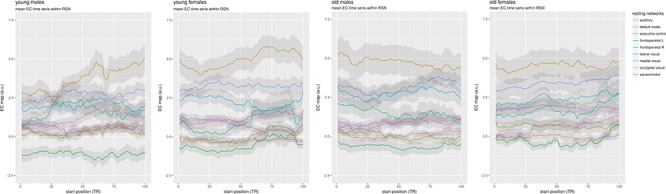
Mean time courses per resting network. Average time series were made from the spatial regression maps minus the cerebellum, which were then averaged per group using a winsorized mean.

Plots of the group mean network time courses found during dual regression were made by averaging the result of the spatial regressions (yielding a time course per network mask) of subjects by group, and then plotting the winsorized mean time course for each network (with a trim of 0.1), and the standard deviation divided by 5 as a ribbon to show the relative variability inside the group for each network (see Figure 4).

Although no obvious difference is visible between males and females, the older groups on the right show less EC variability than the younger groups on the left. There is a partial ordering in the networks in average centrality that is consistent across groups: the DMN has the highest mean centrality in all four groups, followed by the medial and lateral visual networks. The right frontoparietal and occipital visual networks are generally next, with the sensorimotor network being in different positions around them. The auditory and executive control networks are below those. Interestingly, the left frontoparietal network, consistently consistently has the lowest mean EC in all groups, showing a large difference with its right counterpart.

## Discussion

The method of dynamic voxelwise brain network centrality computed from fMRI combines the advantage of ECM of being able to investigate functional brain network properties on the voxel level so that the results can be presented together with other brain image statistic maps, and the sensitivity to time-dependent changes of sliding-window correlation-based analyses of fMRI experimental data.

Comparisons of single-window ECM (Figure 1) with the mean ECM taken over all windows (Figure 2), and their comparison with single ‘static’ ECM from previous studies (Binnewijzend et al., 2014; Duinkerken et al., 2017; Eijlers et al., 2017) show that the pattern of centralities is relatively stable across windows; changes over time are mainly fluctuations around the pattern represented by the mean ECM. This is also in line with the dominant patterns found previously in EC time series (Preti and Ville, 2017).

In line with earlier findings in resting fMRI data network analysis (Achard et al., 2006; Kiviniemi et al., 2011), we found slowly varying variability in our centrality maps. Other methods for studying functional brain network dynamics (Fraiman et al., 2009; Allan et al., 2015; Thompson et al., 2018) find different spatiotemporal dynamics. As our method uses a sliding window, slow temporal variability is expected (Hindriks et al., 2016).

Differences in resting fMRI connectivity between males and females have been described before (Gong et al., 2011) but gender differences in the stability/rigidity of functional brain networks have had less attention. Earlier studies have found lower connectivity with the primary visual and auditory cortex in female participants (Filippi et al., 2013; Smith et al., 2014), indicating that their strength is lower than in men; but this does not mean that centrality in these networks is lower. Synaptic density in the visual cortex has been reported to be higher in male subjects than female (Alonso-Nanclares et al., 2008), although it is not immediately clear that this would lead to stronger covariations within the visual cortex.

Not much is known about resting between-gender functional connectivity differences in the motor network. Ritchie et al. (2018) found stronger functional connectivity in males than in females between the sensorimotor network and visual and prefrontal areas. This could indicate an increased centrality, which could in turn lead to increased temporal correspondence in centrality variations within the motor network. Another study found higher fractional amplitudes of low-frequency fluctuations (fALFF) in the sensorimotor cortex in males than females (Biswal et al., 2010). These slower oscillations may cause a more stable temporal behavior in signal, connectivity, and centrality.

The effect of age on resting-state connectivity has been studied extensively, although this has concentrated more on older subjects. The regions in the first network that shows differences between the age groups in temporal EC variability, medial visual (Figure 3a), have been previously reported as having decreased centrality in patients with Alzheimer’s disease (AD) compared with controls (Binnewijzend et al., 2014) and in subjects with elevated risk factors for AD, including age (Wink et al., 2018). If synchronization in these regions with the rest of the visual network deteriorates, this may cause a lower covariability with the centrality of the rest of the network as well. The fact that an age effect can already be measured in the young adults included here, confirms previous findings of the age-sensitivity of eigenvector centrality in the visual cortex.

Decreased connectivity with age has been measured in the DMN (Vidal-Piñeiro et al., 2014; Sala-Llonch et al., 2015). Geerligs et al. (2015) report age-related changes in the same networks found in this study, where segregation of networks is higher in younger participants: the networks can more clearly be separated. The DMN and the frontoparietal network showed a reduction in local efficiency, which me be related to our intra-network reductions in connectivity and centrality with age. A difference was also found in visual and motor networks between the ‘participation index’ between younger and older subjects, that is: the proportion of signal that is directed toward other RSN. This was higher in older subjects. Our findings may relate to this, in that better concordance with external networks may lead to weaker intra-network connectivities and centralities.

Older adults have also been shown to use the fronto-parietal network more ‘efficiently’ in cognitive tasks to reduce the influence of external distractions, and this network showed lower connectivity at rest (Campbell et al., 2012, 2013). There is also evidence that connectivity between the DMN and frontoparietal networks is an indication of cognitive flexibility (Douw et al., 2016), suggesting mental agility but also distractability, which would decrease with age. However, this combination of terminology is speculative at the moment.

Mean network-specific centralities as measured with the sliding-window approach (Figure 4) show smooth and slow dynamics, indicating that they are relatively stable on the time scale of an fMRI experiment, in agreement with earlier findings on time-varying centrality (Liao et al., 2015), indicating that functional networks are associated with structural connections. This is also reflected by the fact that relative mean network centralities are stable within and between age and gender groups, with the DMN having the highest average centralities in all cases.

The differences in dynamic eigenvector centralities between gender and age groups enjoin graph theoretic analyses of fMRI data studies to model or correct for these factors.

### Limitations

One of the aims of this paper was inviting others to reproduce these analyses. For this reason, we used a publicly available data set with fully pre-processed images to minimize differences in outcomes to pre-processing. A disadvantage of this strategy is the reduced level of control over these previous steps (such as the correction of motion effects between realignment and standard space mapping).

Not all subjects were scanned for the same amount of time, which yields variable underlying signal to noise ratios for the volumes of different ECM time series. At the moment it is not known how this influences the ECM time series dynamics; this is the subject of ongoing work.

The connections matrix used for ECM is the correlations matrix, increased with 1 to guarantee non-negativity (Wink et al., 2012). This equation, which negatively correlates with the squared L2-norm of signals with a Gaussian distribution, cannot be as trivially used for more advanced measures of connections, such as partial correlation (Zhen et al., 2007).

The statistical tests between groups are corrected for multiple voxel comparisons, but not for multiple contrasts (resting networks). If this correction is applied (*p* = 0.05/10) then none of the RSNs show significant differences in dynamics.

## Conclusion

We have presented dynamic ECM, provide an implementation in the fastECM package and demonstrate its application in a publicly available dataset, demonstrating changes in EC variability inside resting networks between gender and age groups.

## Data Availability

The datasets generated for this study are available on request to the corresponding author.

## Author Contributions

AW performed the analyses and wrote the manuscript.

## Conflict of Interest Statement

The author declares that the research was conducted in the absence of any commercial or financial relationships that could be construed as a potential conflict of interest.

## Footnotes

1 https://openneuro.org

2 https://www.fil.ion.ucl.ac.uk/spm

3 https://github.com/amwink/bias/tree/master/matlab/fastECM
